# Baicalin Attenuates Subarachnoid Hemorrhagic Brain Injury by Modulating Blood-Brain Barrier Disruption, Inflammation, and Oxidative Damage in Mice

**DOI:** 10.1155/2017/1401790

**Published:** 2017-08-24

**Authors:** Xianqing Shi, Yongjian Fu, SongSong Zhang, Hao Ding, Jin Chen

**Affiliations:** Intensive Care Unit, Guizhou Province People's Hospital, Guiyang, Guizhou Province 550002, China

## Abstract

In subarachnoid hemorrhagic brain injury, the early crucial events are edema formation due to inflammatory responses and blood-brain barrier disruption. Baicalin, a flavone glycoside, has antineuroinflammatory and antioxidant properties. We examined the effect of baicalin in subarachnoid hemorrhagic brain injury. Subarachnoid hemorrhage was induced through filament perforation and either baicalin or vehicle was administered 30 min prior to surgery. Brain tissues were collected 24 hours after surgery after evaluation of neurological scores. Brain tissues were processed for water content, real-time PCR, and immunoblot analyses. Baicalin improved neurological score and brain water content. Decreased levels of tight junction proteins (occludin, claudin-5, ZO-1, and collagen IV) required for blood-brain barrier function were restored to normal level by baicalin. Real-time PCR data demonstrated that baicalin attenuated increased proinflammatory cytokine (IL-1*β*, IL-6, and CXCL-3) production in subarachnoid hemorrhage mice. In addition to that, baicalin attenuated microglial cell secretion of IL-1*β* and IL-6 induced by lipopolysaccharide (100 ng/ml) dose dependently. Finally, baicalin attenuated induction of NOS-2 and NOX-2 in SAH mice at the mRNA and protein level. Thus, we demonstrated that baicalin inhibited microglial cell activation and reduced inflammation, oxidative damage, and brain edema.

## 1. Introduction

The outcome of hospital admission due to aneurysmal subarachnoid hemorrhage (SAH) is very poor [1]. The major reason for mortality is poor clinical grade during admission, age, aneurysm rebleeding, and vasospasm-associated cerebral infarction [2]. Most studies on SAH were addressed on vasospasm due to the fact that it can significantly hinder blood flow, which correlates with ischemic brain as well as infarction [3]. Prevention of such mortality and development of new therapies depend on understanding the molecular events after SAH. SAH-associated brain injury is a complex process involving inflammation, brain edema, microglial cell activation, oxidative damage, and blood-brain barrier disruption [4]. Each molecular event is crucial and thus can regulate the outcome of the brain injury.

The animal model for SAH is well established, and numerous studies are reported in mice [5]. Recently, few plant-derived natural active ingredients mediated protection in brain injury after SAH has been reported [6–8]. Traditional Chinese medicines have been used for the treatment of various types of diseases for over thousands of years. One of the active ingredients in traditional Chinese medicine, baicalin, is widely used for inflammatory diseases including brain inflammation [9–11]. Pharmacokinetics studies show that baicalin is quickly absorbed and has longer stability in the plasma for over 12 hours [12, 13], thus emerging as a multitherapeutic agent [14].

In this study, we demonstrated baicalin attenuated brain injury after SAH in multistep mechanisms including microglial cell activation, inflammation, modulation of blood-brain barrier function, and oxidative damage.

## 2. Method

### 2.1. Animal Ethical Approval

This study received permission from the Animal Care and Research Committee of Guizhou Province People's Hospital. Adult male C57BL/6 mice (25–30 g) were provided by the animal center of Guizhou Medical University (approval number SCXK (Guizhou) 2002-0001, Guiyang, China).

### 2.2. Animal Surgery Model

We performed the SAH model in mice as described earlier [15]. We observed 31% mortality consistent with published method [15]. Baicalin was administered 15 minutes after surgical procedure at a dose of 100 mg/kg intraperitoneally (i.p.). Sham-operated mice administered with either vehicle (described as control throughout the study) or baicalin at a dose of 100 mg/kg intraperitoneally (i.p.) underwent the same procedure but were not subjected to perforation.

### 2.3. SAH Grade Assessment

SAH grade by bleeding scale in filament perforation subarachnoid hemorrhage was performed according to a previously described 18-point-score method [16]. In brief, the basal cistern was divided into 6 segments and the subarachnoid blood clots are assessed in each of these segments to allot a score from 0 to 3.

### 2.4. Neurologic Score

Neurologic score was determined as described previously [17]. This is an 18-point Garcia scale and a 4-point balance beam test with slight modification. Six tests, namely, spontaneous activity, spontaneous movement of four limbs, forepaw stretching, climbing, proprioception, response to whisker stimulation (score 3–18), and adding balance beam test (score 0–4). All experiments were performed by two blinded researchers.

### 2.5. Tissue Collection and Brain Water Content (Brain Edema)

The animals were sacrificed under deep anesthesia (5% isoflurane), and brains were sectioned as brain stem, left and right hemisphere, and cerebellum. The tissues were weighed under wet condition followed by reweighing after drying at 105°C for 24 hours. The percentage of water content was calculated as ([wet weight − dry weight]/wet weight) × 100% as described earlier [18].

### 2.6. Quantitative Real-Time PCR

Total RNA was extracted from whole brain tissue (left and right hemisphere) according to the manufacturer's protocol (Qiagen RNeasy Mini Kit, Qiagen, Hilden, Germany). Total RNA was reverse transcribed into cDNA by Qiagen Reverse Transcription Kit (Qiagen, Hilden, Germany). Real-time PCR was carried out using 7500 Real-Time PCR system (Applied Biosystems) with 200 pM PCR primers. Each sample was analyzed using SYBR green (Applied Biosystems) as fluorescent detector and actin as endogenous control. All primers were obtained from RT qPCR Primer assay kits (SAB Bioscience). The data were analyzed and expressed as fold using comparative Ct method.

### 2.7. Immunoblot

Proteins from perforation side tissue samples were isolated by lysing in RIPA buffer with protease inhibitor cocktail (Roche). The proteins were quantified with bicinchoninic acid protein assay kit (Beyotime Biotech, China). Equal amount of proteins were loaded onto gradient PAGE, transferred into nitrocellulose membrane. After blocking with 10% nonfat milk in PBS-Tween20, membranes were incubated with antibodies IBA1, GAPDH, p65, histone H3, NOS2, and NOX2 (Abcam China, Shanghai, China). After repeated washing, the membrane was incubated with corresponding secondary antibodies (Beyotime, Shanghai, China) followed by chemiluminescence detection.

### 2.8. Microglia Cell Isolation and Cell Culture

Microglial cells from heathy adult mouse were isolated as described before [19]. Cells were plated onto cell culture flasks in Dulbecco's modified Eagle's medium/Ham's F-12 (DMEM/F12, Life Technologies) with 10% fetal bovine serum (FBS, Life Technologies). Lipopolysaccharide at 100 ng/ml was added for 24 hours, and culture supernatant were quantified for IL1*β* and IL6 cytokine using Quantikine ELISA kit (R&D System, USA). The cell pellet was processed with N-PER nuclear extraction kit for Western blot experiments.

### 2.9. Statistics

All values are expressed as the mean standard derivation. One-way analysis of variance followed by Tukey's multiple comparisons test was used for comparisons between two groups, and c2 tests were used for behavior score analyses. A minimum *p* value < 0.05 was considered statistically significant.

## 3. Results

### 3.1. Baicalin Improved Neurological Score and Brain Edema in SAH Mice

**Figure 1:**
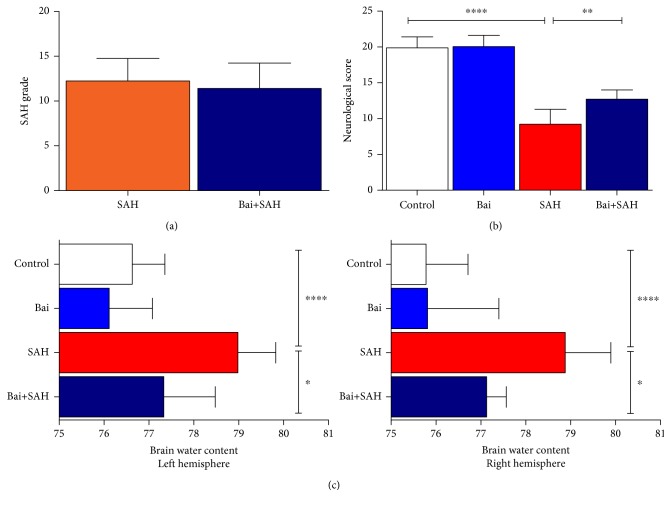
Neurological score and brain edema 24 hours after SAH. (a) SAH grading scores in SAH with either vehicle or baicalin posttreatment groups. (b) Neurological score for each group 24 hours after SAH. Values are mean ± standard deviation. *N* = 6 in each group, ∗∗∗∗ represents *p* < 0.0001, and ∗∗ represents *p* < 0.005. (c) Effect of baicalin on brain water content in the left and right hemispheres 24 hours after SAH. *N* = 6 in each group, ∗∗∗∗ represents *p* < 0.0001, and ∗ represents *p* < 0.05.

Neurological score was determined 24 hours after SAH surgery. There was no significant difference in SAH grade between SAH with vehicle or baicalin treatment group (Figure 1(a)). However, neurological scores were significantly lower in SAH groups compared to control or baicalin-treated sham-operated mice. Baicalin-treated mice improved in neurological score compared to vehicle-treated SAH mice, and it was statistically significant (*p* = 0.004) (Figure 1(b)). Brain edema as determined by brain water content was examined. As reported previously, SAH significantly increased water content in both left and right hemispheres. Baicalin treatment reduced brain water content in both hemispheres in a statistically significant manner (*p* = 0.025 for the left and *p* = 0.043 for the right) (Figure 1(c)).

### 3.2. Baicalin Attenuated Increased Blood-Brain Barrier (BBB) Permeability and Tight Junction Protein Degradation in SAH Mice

**Figure 2:**
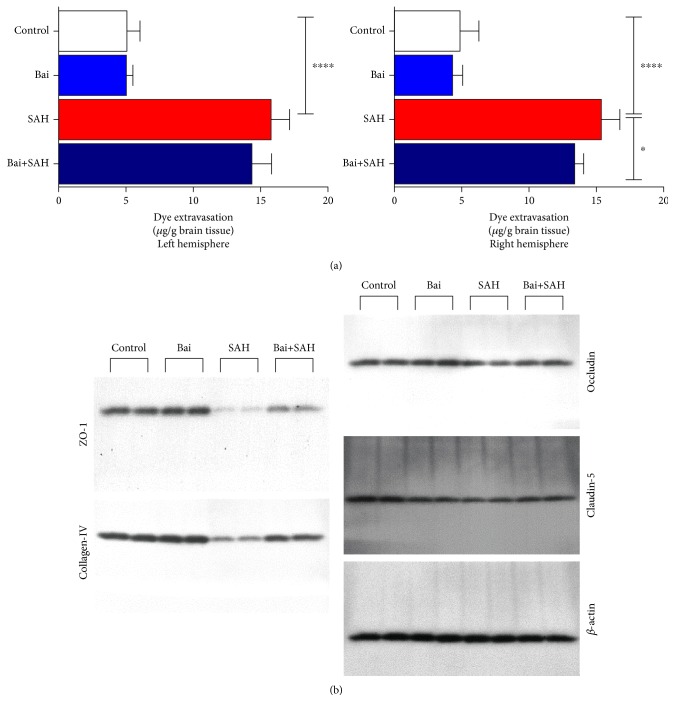
Effect of baicalin on BBB permeability and tight junction proteins in SAH mice. (a) Evans blue dye extravasation was observed in each set after SAH. The result demonstrated that BBB permeability was increased and attenuated by baicalin. Values are mean ± standard deviation. *N* = 6 in each group, ∗∗∗∗ represents *p* < 0.0001, and ∗ represents *p* < 0.05. (b) Western blot analyses of occludin, claudin-5, ZO-1, and collagen IV.

Blood-brain barrier (BBB) function is critical as SAH increases its permeability [20]. We used Evans blue extravasation assays to determine BBB permeability. SAH induced permeability. Baicalin treatment restored BBB function to normal level (Figure 2(a)). The effect was significant in the right hemisphere (*p* < 0.05), whereas a similar trend was observed in the left hemisphere. Tight junction transmembrane proteins such as occludin and claudin along with ZO-1 proteins have a crucial role in maintaining blood brain barrier function [21]. These proteins were degraded in SAH and thus contribute to permeability increase. We observed a decrease in protein level of occludin, claudin-5, ZO-1, and collagen IV by SAH. Baicalin treatment restored the protein levels close to normal (Figure 2(b)).

### 3.3. Baicalin Attenuated Production of Inflammatory Cytokines and Inflammation in SAH Mice

**Figure 3:**
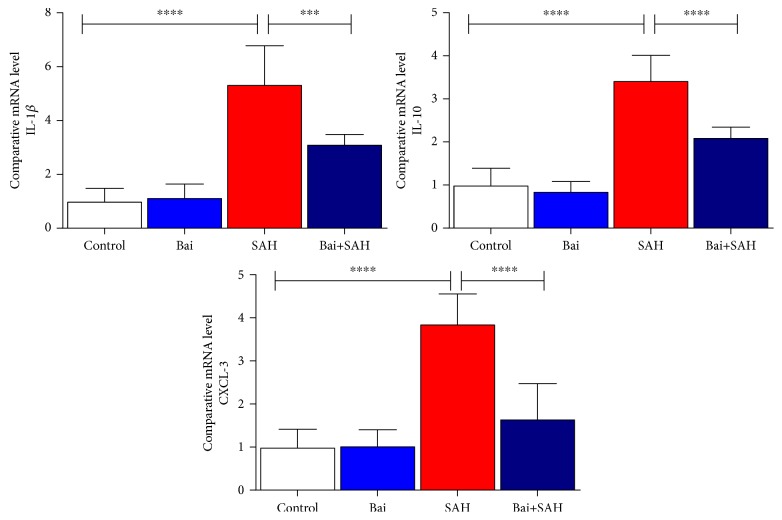
Effect of baicalin on proinflammatory cytokine mRNA expression in SAH mice. Real-time PCR analyses of IL-1*β*, IL-6, and CXCL-3. Values are mean ± standard deviation. *N* = 6 in each group, ∗∗∗∗ represents *p* < 0.0001, and ∗∗∗ represents *p* < 0.0005.

**Figure 4:**
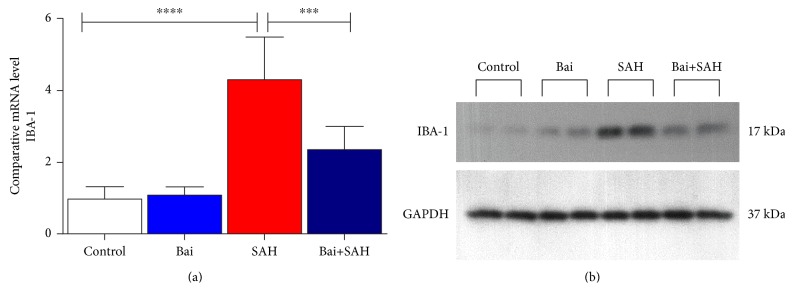
Effect of baicalin on glial cell activation marker IBA-1 expression in SAH mice. (a) Real-time PCR analyses of IBA-1. Values are mean ± standard deviation. *N* = 6 in each group, ∗∗∗∗ represents *p* < 0.0001, ∗∗∗ represents *p* < 0.0005. (b) Immunoblot analyses of IBA-1 along with loading control GAPDH.

Release of cytokines particularly IL-1*β* has a critical role in early brain injury of SAH mice [20]. We determined three cytokines, IL-1*β*, IL-10, and CXCL-3, by real-time PCR in total brain samples. All three cytokines were increased 24 hours after SAH. Baicalin treatment attenuated significantly IL-1*β*, IL-10, and CXCL-3 cytokine mRNA levels (Figure 3). To address further, we also examined the amount of inflammation using microglial cell marker IBA-1 by real-time PCR (Figure 4(a)) and immunoblotting (Figure 4(b)). Both data were consistent with an earlier finding that the increase of glial cell population (inflammatory response) occurred after SAH brain injury. Baicalin attenuated inflammatory response significantly.

### 3.4. Baicalin Attenuated Glial Cell Activation In Vitro

**Figure 5:**
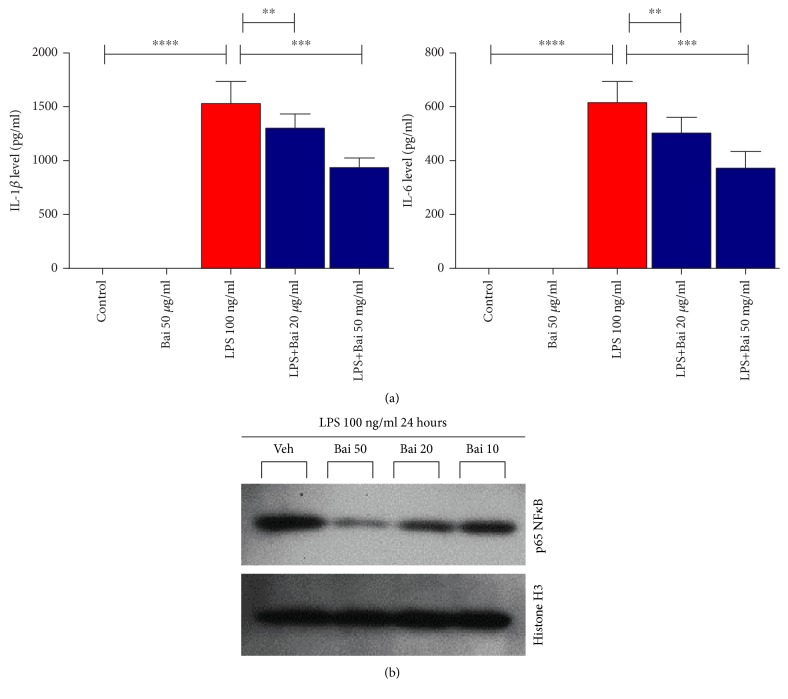
Effect of baicalin on glial cell activation in vitro. (a) Baicalin dose dependently inhibited the secretion of proinflammatory cytokines IL-1*β* and IL-6, which were determined by ELISA. *N* = 6 in each group, ∗∗∗∗ represents *p* < 0.0001, and ∗∗∗ represents *p* < 0.0005. (b) Immunoblot analyses of p65 from nuclear fraction along with loading control histone H3. ∗∗ represents *p* < 0.005.

We investigated primary microglial cells for activation by LPS and protection by baicalin. Primary microglial cells were stimulated with LPS (100 ng/ml). Pretreatment with baicalin dose dependently inhibited secretion of proinflammatory cytokines IL-1*β* and IL-6 (Figure 5(a)). LPS induced nuclear localization of p65 NF*κ*B in primary microglial cells and was attenuated by baicalin in a dose-dependent manner (Figure 5(b)).

### 3.5. Baicalin Attenuated Induction of NOS-2 and NOX-2 in SAH Mice

**Figure 6:**
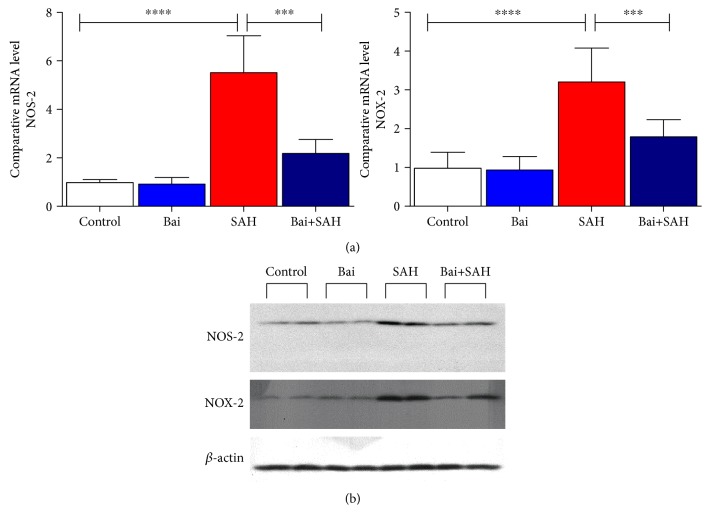
Effect of baicalin on NOS-2 and NOX-2 mRNA expression in SAH mice. (a) Real-time PCR analyses were performed on two oxidative stress-generating enzymes NOS-2 and NOX-2. Values are mean ± standard deviation. *N* = 6 in each group, ∗∗∗∗ represents *p* < 0.0001, and ∗∗∗ represents *p* < 0.0005. (b) Immunoblot analyses of NOS-2 and NOX-2 from total brain lysates with loading control beta-actin.

Glial cell activation is associated with NOS-2 (iNOS) and NOX-2 induction [22–24] in brain injury. These two enzymes in glial cell lead to oxidative damage in the brain [25]. In SAH mice, both NOS-2 and NOX-2 were induced and baicalin attenuated induction of both enzymes in a statistically significant level as evidenced by real-time PCR (Figure 6(a)). The mRNA data were further confirmed by immunoblot analyses (Figure 6(b)).

## 4. Discussions

Our evaluation on the effect of baicalin on SAH in mice demonstrated that baicalin reduced proinflammatory cytokine production and oxidative damage-causing sources. SAH is a subtype of stroke where the mortality is very high and the survivors also have difficultly in the long term. The first 72 hours is a critical time for all SAH patients. Most clinical cases are caused by rupture of intracranial aneurysms located in the brain arteries [26]. It is technically difficult to produce aneurysms to study in animal models, but a previously published method of filament perforation model for SAH in mice demonstrates in a standardized and reproducible manner [15]. We demonstrated by SAH grading that the data was reproducible among all samples. However, the posttreatment of baicalin did significantly affect neurological scores. It is recently reported that baicalin administration reduces infract volume in mouse cerebral ischemia model [27]. Thus, the effect of baicalin in brain injury is beneficial.

An increase in BBB permeability after SAH has been reported in patients and animal models [28]. The difference in BBB disruption of the intraparenchymal vessels after SAH may have possible pathophysiologic implications in relation to brain edema and microcirculatory disturbances occurring during the early clinical course of patients suffering with SAH [29]. Our data demonstrated that baicalin restored SAH-mediated BBB function significantly in the right hemisphere, and a similar trend was observed in the left hemisphere. This effect might be due to the fact that baicalin also modulated glial cell activation and their population difference in the two hemispheres.

**Figure 7:**
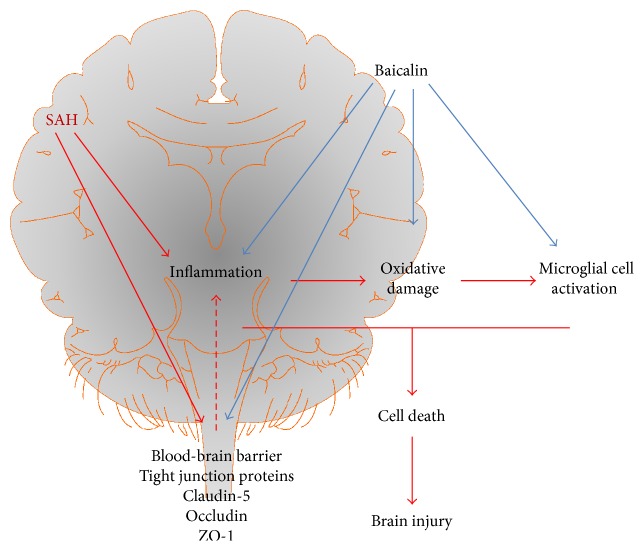
Schematic diagram of baicalin-mediated protection in SAH mice. SAH injury in brain leads to three physiological responses, namely, inflammation, microglial cell activation, and oxidative damage, which lead to cellular injury and cell death. Baicalin modulates all three physiological processes and thus attenuated SAH-mediated brain injury. Baicalin also restores blood-brain barrier function impaired by SAH, where degradation of tight junction proteins was modulated.

Inflammation plays a critical role in brain injury after SAH [30]. The early release of inflammatory cytokines is also associated with brain edema [31]. In our study, we observed that baicalin attenuated proinflammatory cytokine production and also associated brain edema (Figure 7). Glial cells in microglia modulate microcirculatory blood flow and synaptic plasticity [32]. After release of proinflammatory cytokines in microglia, glial cell activation occurs. After activation, those cells quickly change to phagocytose any unwanted particles and secrete more proinflammatory cytokines to facilitate an immune response [33]. We have demonstrated that baicalin can modulate microglial cell activation *in vitro* (Figure 7). This is a critical step to contain brain injury and oxidative damage as severe immune response leads to edema and more oxidative damage-associated neuronal cell death. Baicalin is effective in many other inflammatory disorders [34–36].

NF-*κ*B is crucial for glial cell function and regulates many genes, which encode important protein players involved in immune function and inflammation [37]. NF-*κ*B plays a critical role in the early stage of SAH [38]. We also observed a similar pattern in our model, and baicalin attenuated the process. Experimental data suggest that a cascade of physiological events occur very early after the initial insult associated with SAH, causing a long-term cognitive deficit [39]. Our data demonstrate that the beneficial effect of baicalin at early injury can have a therapeutic benefit in the long term, and further experiments are required to assess that.

NOS-2 and NOX-2 are implicated in oxidative damage of subarachnoid hemorrhagic brain injury [40–42]. We observed baicalin attenuated induction of both enzymes. NOS-2 is a major generator of nitric oxide, which leads to deadly peroxynitrite radicals [43, 44]. Oxidative damage is responsible for a poor prognosis of SAH patients. NOX-2, which generates superoxide radical, has been present in perihematomal neurons and astrocytes of SAH animals [45]. Thus, our study indicated that baicalin-mediated reduction of both enzymes in SAH attenuated oxidative damage and cell death (Figure 7).

Traditional herbal medicine is considered to be the bench mark for multitarget therapy with promising future [46]. Baicalin, which is also part of many traditional Chinese medicines, has been demonstrated to have a potential role in many tissue injury animal models including liver, kidney, heart, pancreatitis, and brain [34, 47]. Baicalin is studied for robust pharmacokinetics and is a potential candidate for drug development [14].

## 5. Conclusion

We provided first preclinical evidence that baicalin protected against subarachnoid hemorrhagic brain injury. The process of attenuation was mediated through modulation of inflammation, brain water content, improving tight junction proteins, microglial cell activation, and oxidative damage. Additionally, we demonstrated that baicalin inhibited lipopolysaccharide-induced activation of primary microglial cells. However, a future study for detailed mechanism including M1/M2 polarization of microglial cells by baicalin may lead to potential new therapeutic development.
